# Identification of QTLs for rice grain size and weight by high-throughput SNP markers in the IR64 x Sadri population

**DOI:** 10.3389/fgene.2022.955347

**Published:** 2022-08-19

**Authors:** Kashif Aslam, Shahzad Amir Naveed, Muhammad Sabar, Ghulam Shabir, Shahid Masood Shah, Abdul Rehman Khan, Muhammad Musaddiq Shah, Sajid Fiaz, Jianlong Xu, Muhammad Arif

**Affiliations:** ^1^ National Institute for Biotechnology and Genetic Engineering (NIBGE), Faisalabad, Pakistan; ^2^ Institute of Molecular Biology and Biotechnology, BZ University, Multan, Pakistan; ^3^ National Institute for Genomics and Advanced Biotechnology (NIGAB), Islamabad, Pakistan; ^4^ Rice Research Institute, Kala Shah Kaku, Pakistan; ^5^ Department of Biotechnology, COMSATS University Islamabad, Abbottabad, Pakistan; ^6^ Department of Biological Sciences, International Islamic University Islamabad, Islamabad, Pakistan; ^7^ Department of Plant Breeding and Genetics, The University of Haripur, Haripur, Pakistan; ^8^ Shenzhen Branch, Guangdong Laboratory for Lingnan Modern Agriculture, Agricultural Genomics Institute at Shenzhen, Chinese Academy of Agricultural Sciences, Shenzhen, China; ^9^ Institute of Crop Sciences, National Key Facility for Crop Gene Resources and Genetic Improvement, Chinese Academy of Agricultural Sciences, Beijing, China

**Keywords:** grain weight, QTL mapping, SNP markers, grain size, genotyping

## Abstract

Grain appearance is one of the most important attributes of rice. It is determined by grain size, shape, and weight, which in turn influences the rice yield and market value. In this study, QTLs for grain length, grain width, grain length/width ratio, and grain weight were mapped using the high-throughput indica/indica SNP platforms. The population of the mega indica variety IR64 and the high-quality aromatic variety Sadri from Iran was phenotyped. Based on this phenotypic data, plants of 94 F_2:3_ families including both parents were selected. A linkage map analysis of 210 SNP markers identified 14 QTLs controlling the grain length, grain width, length/width ratio, and 1,000 grain weight. Among these 14, one important region containing the QTLs for all the four studies’ traits was mapped on chromosome 8. It was derived from Sadri for the decreased length/width ratio and increased grain weight. This study demonstrated the speed and efficiency in using multiplex SNP genotyping for QTL analysis. Moreover, this study identified four novel QTLs (qGL8, qTGW8, qLWR8, and qGW8) sharing the same position on chromosome 8 which were linked with grain quality characteristics between one indica and one aromatic variety. It will enable more precise marker-assisted selection for grain weight, shape, and size. Further in-depth studies are required to dissect this region of interest and identify the related gene(s).

## Introduction

Rice is one of the most important cereal crops. The grain quality of rice has several components: biochemical characteristics, cooking quality, flavor, milling efficiency, grain shape, appearance, and nutritional value. Among these, grain size (length and width), grain shape (length/width ratio), and weight are the main components of yield. Grain size is becoming target breeding traits for improving the rice yield (Xing and Zhang, 2010). Grain appearance is also important to capture the higher market value of rice. Global preferences for grain size and grain shape vary widely, some preferring long and cylindrical grain is preferred in some regions (e.g., United States and Europe), while short and round grain is preferred in other parts of the world (e.g., China, Japan, and Korea) (Bai et al., 2010). Across large regions of South East Asia, IR64 has been widely grown and accepted by consumers for desirable grain quality. Likewise, in some countries, such as Iran, consumers prefer aromatic varieties of rice such as the Sadri. The grain shape and size are quantitative traits (McKenzie and Rutger, 1983), and several individual studies have identified a number of QTLs for grain size and grain weight (Tan et al., 2000; Wan et al., 2008; Huang et al., 2013; Nagata et al., 2015; Wang et al., 2015; Gao et al., 2016; Cabral et al., 2018; Wu et al., 2020). A large number of grain length QTLs were reported by Wan et al*.* (Shao et al., 2010) and Zhang et al. (Yan et al., 2011) on chromosomes 2, 3, 5, 7, and 9 explaining the percentage of phenotypic variation ranging from 5.8 to 35.6 in three different environments based on SSR and EST markers in the cross of japonica (IR24)/Japonica (Asominori). Six QTLs were reported by Wan et al. (2008) which affect grain width in four different environments and found qGW5 as the major QTL on chromosome 5 with phenotypic variation ranging from 22.2 to 25.9% in different environments using F_7_ RILs derived from indica and japonica cross. Liang-Qiang et al. (2006) detected the gene Lk-4 (t) controlling the grain length of chromosome 4 with the help of SSR and CAPs markers in BC_2_F_2_ population derived from Shuhui527 and Xiaoli. Two QTLs were also mapped on chromosome 7, in two independent studies using populations derived from indica and japonica varieties, qGL7 (Bai et al., 2010) and qGL7-2 (Shao et al., 2010). Recently, many QTLs have been cloned for grain shape and size such as GS3, GW2, and qGW5 (Yan et al., 2011). The major gene GS3 identified on the precentromeric region of chromosome 3 was shown to have a major effect on grain length and weight whereas a minor effect on grain weight, the results showed that all these traits are correlated (Fan et al., 2006). Song et al. (2007) identified a gene GW2 on chromosome 2, which encodes a RING-type protein with E3 ubiquitin ligase activity controls grain width and grain weight. Yan et al. (2011) found that GW2 and qGW5 positively regulate the expression of GS3. The small and round seed1 (SRS1) gene was identified as effecting grain size, which is the same as previously reported gene dense and erect panicle 2 (DEP2) (Abe et al., 2010). Molecular markers, especially SSRs, have been successfully used to identify gene/QTLs for complex traits across a variety of germplasm. However, there are some limiting factors because SSR markers can have multiple alleles and it is difficult to multiplex the SSR markers to screen large populations. SSR marker results also vary by laboratory, germplasm, and population size because of its multiallelic nature causing a higher probability of detecting heterozygosity (Lee et al., 2022) [98]. Additionally, SSR markers are difficult to align a diverse array of alleles in the database because the rate of mutation is much higher (10–10,000 times more frequent than single nucleotide substitutions per generations (Morales et al., 2020) [99]). It is because with the intervention of the technological advances, millions of bi-allelic markers have been identified in the rice genome as single-nucleotide polymorphic (SNP) markers, allowing the construction of high-quality genetic linkage maps and to identify and fine map of QTL/genes (McCouch et al., 2010). SNP markers have improved the methods for QTL/gene identification and have recently been widely used in marker-assisted selection (MAS) (Phing Lau et al., 2016), genome-wide diversity, and association analysis (McCouch et al., 2010). SNP markers are easy to multiplex, which reduces time and cost. To apply these markers, different platforms have been developed with different sets in terms of SNP marker density, such as 96, 384, 1536, and 44,000-plex (McCouch et al., 2010; Thomson et al., 2021). All of these SNP markers are bi-allelic, so results from different studies can be easily combined and shared between laboratories. For the Illumina BeadXpress reader, 94 and 384-plex SNP sets were used to develop a linkage map and identify QTLs for indica/indica, indica/japonica, and japonica/japonica combination (Thomson et al., 2012). Naveed et al. (2018) also reported that SNP markers are a useful tool to identify gene/QTLs controlling complex traits. Populations derived from indica x indica were studied by Niu et al. (2021), and 23 QTLs were detected in all 12 chromosomes except chromosomes 1, 3, and 9. All these studies confirm the significant role of SNP markers in QTL identification. This study was designed with the aim to identify QTLs affecting the grain appearance and grain weight in the population developed from Sadri (aromatic) and IR64 (non-aromatic) with the help of SNP markers by using the Illumina BeadXpress system. In addition to 10 other QTLs, a major QTL with a strong effect on grain length and grain shape was found on chromosome 8 and reported the allele from the donor parent effect the grain length and grain length to width ratio.

## Materials and methods

### Population development

Sadri (ACC 32339) was crossed with IR64 (paternal: pollens were used) at IRRI Philippines. F_1_ and F_2_ populations were advanced by self-fertilization. Phenotypic trait data were measured for each plant in the F_2_ population. In total, 140 F_2_ plants were harvested to measure the grain length and width. These data were recorded by using 30 randomly selected grains with their bran intact through a HP 8200 Digital Flatbed scanner and repeating it three times, and grain images were then analyzed by the computer software application GIMP 2.8 (http://www.gimp.org/) to measure the grain length, width, and length–width ratio. Thousand-grain weights per family at the 12% moisture level were measured using a digital balance. Based on the phenotypic data in F_3_ of these 140 plant progenies, 94 were selected for further molecular analysis.

### DNA extraction

DNA was extracted from these 94 F_3_ progenies for molecular analysis and approximately 100–150 plants of each progeny were pooled together to represent the true genotype of F_2_ progeny. DNA was extracted by using a slightly modified protocol reported by Murray and Thompson (1980). Rice leaves were ground in liquid nitrogen in a mortar and pestle. The powder was placed in 2 ml centrifuge tubes, followed by the addition of 2x CTAB buffer (CTAB 20 g, 100 mM Tris-HCL [pH 8.0], 50 mM EDTA [pH 8.0], and 500 mM NaCl) with 2% SDS, chloroform extraction, and final precipitation by 2-propanol (Murray and Thompson, 1980). DNA was dissolved in 100 μl TE buffer with 1 µl of 10 mg/μl of RNAse. DNA samples were quantified with the help of a NanoDrop spectrophotometer and normalized to 70 ng/μl concentration. The DNA samples were run on 1% agarose gel to check the quality of DNA.

### SNP genotyping by using Illumina BeadXpress

GoldenGate assay through VeraCode technology was used for the genotyping (Manly et al., 2001; Lin et al., 2009). VeraCode technology uses cylindrical microbeads that barcode the unique oligo-nucleotide sequence to corresponding SNP loci which hybridized with the labeled (cy3/cy5 dye) polymerized product (Lin et al., 2009). The SNP markers were designed and prepared by following the “GoldenGate Genotyping Assay for VeraCode Manual Protocol’’ (Illumina Part # 11275211) and manufacturer’s instructions. For population genotyping, 96-well plates with a total volume of 5 µl of 70 ng/μl of the DNA sample were used. Amplification was performed in a G-Strom (Surrey, United Kingdom) thermal cycler through a universal primer amplification step (Thomson et al., 2012). After the amplification step, the BeadXpress reader scanned the VeraCode beads hybridized to each address sequence. For each SNP, homozygous genotypes were represented by a single fluorescent dye, i.e., Cy3 or Cy5, whereas the appearance of both colors in the equal amount at that particular locus indicated the presence of the heterozygous genotype.

### Data analysis

All SNP data generated by BeadXpress were analyzed by Illumina GenomeStudio (v2010. 1) software on the basis of the ratio cy3/cy5 signal intensities, and data were exported by using the ALCHEMY-Illumina plug-in (v1. 0). Following the procedure applied in the Human Genome Project, ALCHEMY software (hhtp://alchemy.sourceforge.net) was used for GenomeStudio to optimize more heterozygous results. Illumina Gene Call software generated the graphical display for each SNP marker (only SNP id4009024 is shown in Supplementary Figure S1). The data points color codes for the call are (AA = red, AB = purple, and BB = blue) based on the ratio of the cy3/cy5 dye signal. Genotypes are called for each sample (dots) by their signal intensity (norm R, y-axis) and allele frequency (Norm Theta, x-axis) relative to the conical cluster position (dark shading) for a given SNP marker (Supplementary Figure S1). ALCHEMY requires three files: 1) a sample format; 2) an SNP intensity report (generated by GenomeStudio); and 3) an SNP map file. A linkage map was developed with the help of Map Manager QTX (Manly et al., 2001), and the file was exported to QTL Cartographer v2. 5 (http://statgen.ncsu.edu/qtlcart/WQTLCart.htm) (Wang, 2006). The data on 94 F_2_ families with 213 SNP markers and four traits were analyzed by Composite Internal Mapping (CIM) with 1000 permutation tests for all traits at the significance level of 0.05. LOD score was set at 3 as a threshold level. R-Cropstat was used for the calculation of the correlation coefficient.

## Results

### Grain size, grain shape, and grain weight phenotypic variation

**FIGURE 1:**
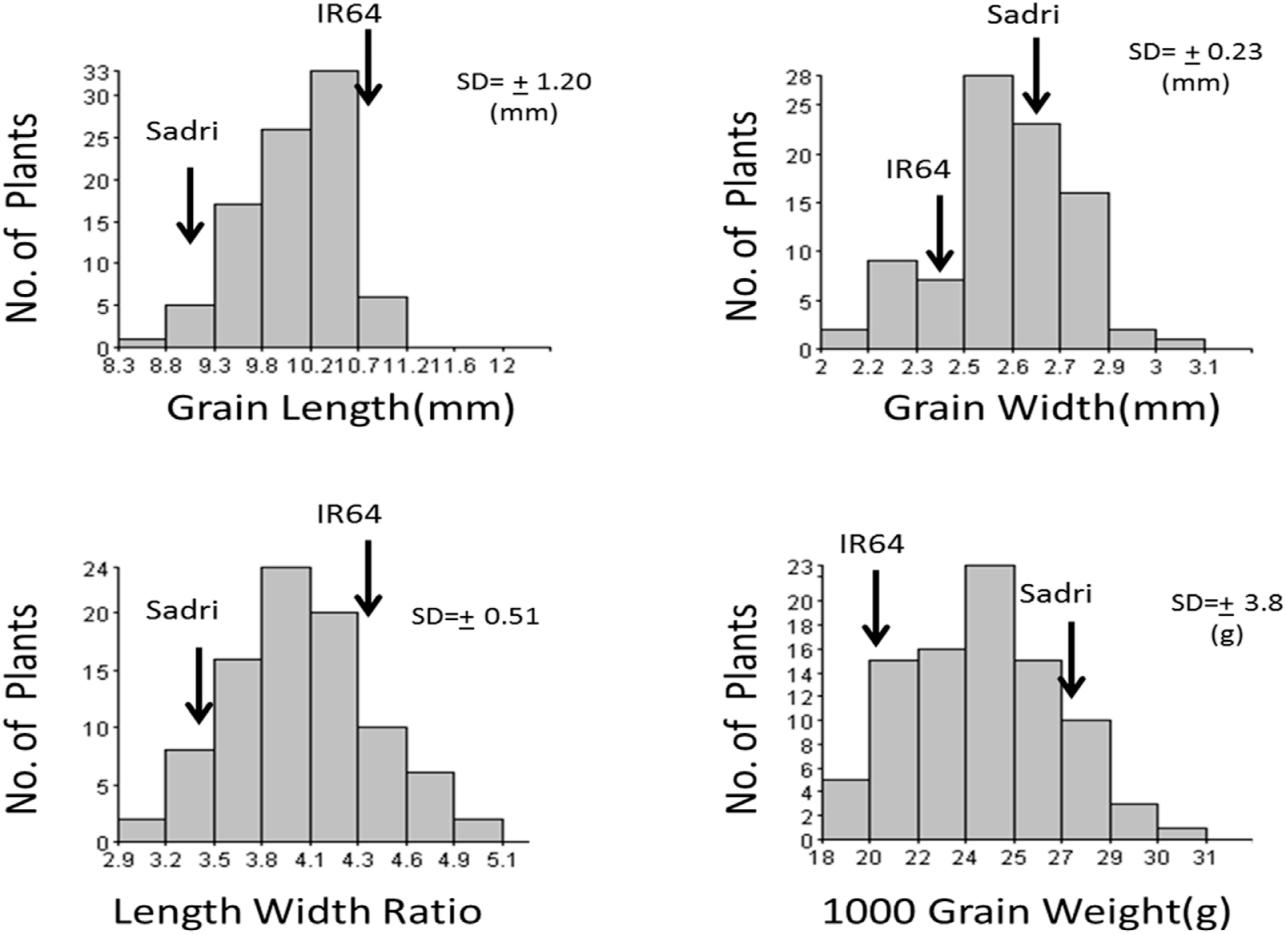
Frequency distribution of grain size, grain shape, and grain weight in F_2_ population.

IR64 has been one of the most popular nonaromatic varieties under cultivation in various parts of the world for several decades for its various characteristics including its long-grain size while Sadri is an aromatic land race cultivated in Iran. Significant differences in all four traits related to grain appearance were recorded between the two parents. F_3_ seeds from 140 F_2_ individuals were phenotyped and showed normal distributions across all four traits (Figure 1). IR64 has a higher trait value for grain length and length/width ratio, whereas Sadri showed higher trait values for grain width and grain weight.

### Correlation coefficient

The correlation coefficient of grain length, grain weight, grain thickness, and thousand-grain weight showed that these traits highly correlated with each other (Table 1). The grain length has a significant negative correlation (−0.32) with the grain width, while it has a significant positive correlation (0.69) with the length–width ratio. Grain length has a positive but minor correlation (0.15) with grain weight (*p* < 0.05). Grain width has a strong negative correlation (−0.89) with the length–width ratio and has a significant positive correlation (0.49) with thousand-grain weight.

**TABLE 1:** Correlation coefficient by Pearson’s product-moment correlation.

	Grain length	Grain width	Length width ratio	1000 grain weight
Grain length	1			
Grain width	−0.32**	1		
Length width ratio	0.69***	−0.89***	1	
1000 grain weight	0.15*	0.49***	−0.31**	1

*, **, and *** significant at levels of α = 0.05, 0.01 and 0.001, respectively.

### Linkage map construction

The rice SNP set of 384-plex named RiceOPA (oligo pool assay) 2.1 was made for indica/indica comparison and RiceOPA3.1 (indica/japonica). Thomson et al. (2012) showed 210 and 190 polymorphic SNPs, respectively, in both parents. Using RiceOPA 2.1, polymorphic markers vary across 12 chromosomes (Chr.) of rice ranging from 9 (Chr. 5) to 29 (Chr. 1) as shown in Table 2. All markers were the same in order according to the physical order of the Nipponbare genome except two markers (wd8001854 and ud8001072) on chromosome 8.

**TABLE 2:** Number of markers per chromosome and chromosome length in centiMorgen (cM).

Chromosome number	Number of markers	Chromosome length (cM)
1	30	259.7
2	21	212.6
3	22	285
4	13	160.3
5	9	72.0
6	13	125.6
7	19	170.7
8	18	126.5
9	15	109.7
10	13	90.2
11	20	199.6
12	17	93.3

### QTL mapping

Using the high-throughput SNP platform BeadXpress, 14 QTLs controlling grain shape, grain size, and grain weight were identified. The QTL cartographer was run with 1,000 permutations at a threshold level of 3.0 LOD. Four QTLs (qGL4, qGL7, qGL8, and qGL11) for grain length (GL) were identified on chromosomes 4, 7, 8, and 11, respectively. Among these QTLs, qGL4 (id4008430-id4009024) had a 3.41 LOD score and explained 3.47% of the phenotypic variation, QTL qGL7 (id7003359-id7003748), with a 4.14 LOD score, explained 8.05% phenotypic variation. Two major QTLs, i.e., qGL8 (id80006789-id8007301) and qGL11 (id11007488-id11008036), with 5.37 and 4.32 LOD scores, explained 29.61% and 16.84% of phenotypic variation, respectively.

Four QTLs (qGW1a, qGW1b, qGW7, and qGW8) for grain width (GW) were detected on chromosomes 1, 7, and 8. Two QTLs, qGW1a (id1024972-id1052983) and qGW1b (id1052983-id1028304) were detected on chromosome 1, having 3.18 and 5.11 LOD and explaining 13.62% and 11.58% phenotypic variation, respectively. The QTL qGW7 (id7002051-id7002105) was identified on chromosome 7, having 3.69 LOD score and explaining 9.77% of the phenotypic variation. The QTL qGW8 (id80006789-id8007301) was identified on chromosome 8 having the highest LOD score of 11.19 and explaining 9.77% of the phenotypic variation.

Three QTLs (qLWR1, qLWR8, and qLWR9) for the length width ratio (LWR) were identified on chromosomes 1, 8, and 9. The QTL qLWR1 (id1000556-id1001073) had a 4.56 LOD score and explained 2.88% of the phenotypic variation. The QTL qLWR8 (id80006789-id8007301), with a 14.55 LOD score, explained 21.90% phenotypic variation. QTL qLWR9 (id9001297-id9001352), with a 3.45 LOD score, explained 5.91% phenotypic variation.

Three QTLs (qTGW1, qTGW3, and qTGW8) for thousand-grain weight (TGW) were identified on chromosomes 1, 3, and 8. QTL qTGW1 (id1024972-id1025983) had a 5.49 LOD score and explained 32.52% of the phenotypic variation. QTL qTGW3 (id3013765-id3014401), with a 3.05 LOD score, explained 8.83% phenotypic variation. QTL qTGW8 (id8006789-id8007301), with a 5.28 LOD score, explained 2.01% phenotypic variation.

**FIGURE 2:**
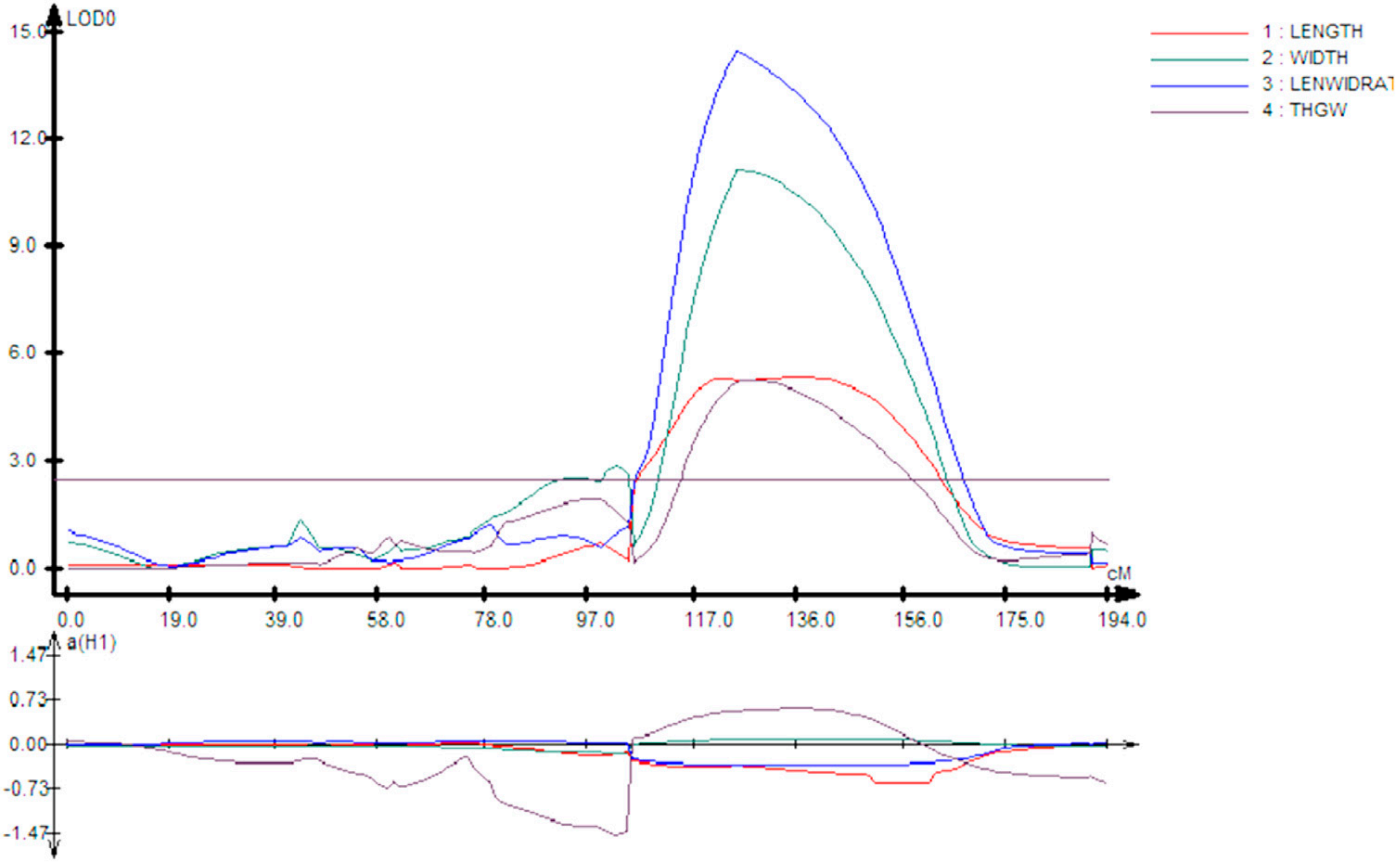
LOD and additive effect of QTLs on chromosome 8 of all four traits.

**FIGURE 3:**
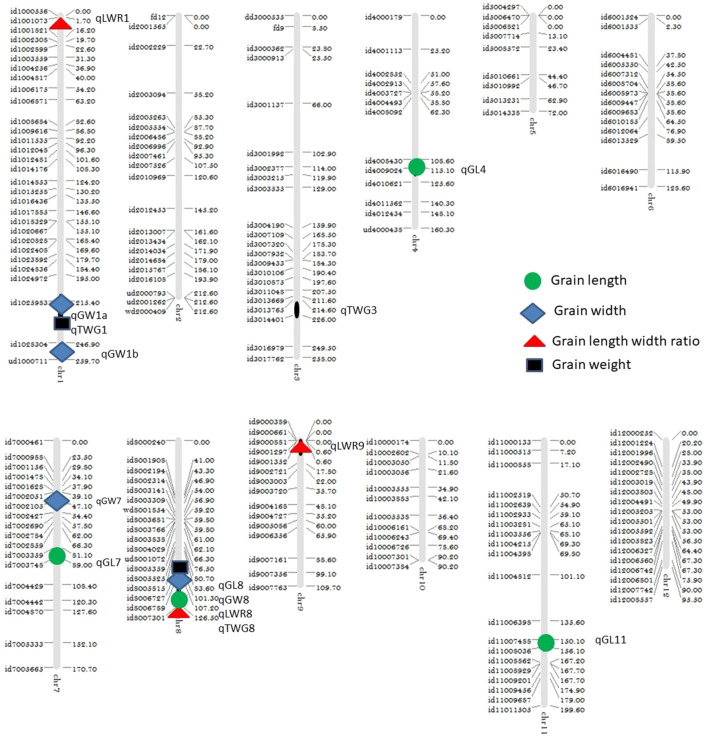
Linkage map showing the QTL location on chromosomes.

Interestingly, chromosome 8 harbor QTLs for all the four traits under consideration. Careful analysis revealed that all these QTLs (qGL8, qGW8, qLWR8, and qTGW8) are located between the marker interval id80006789-id8007301, indicating that this locus is involved in all of these four traits. The LOD score of all traits varies from low 5.37 (qGL8) to 14.55 (qLWR8) as shown in Figure 2. The QTLs identified on the different chromosome are shown in Figure 3 and the LOD, their share in explaining the phenotypic variation, and marker interval are given in Table 3.

**TABLE 3:** QTLs for grain length, grain width, grain length–width ratio, and grain weight.

QTL	Chr	Interval	Physical position (MB)	LOD	Additive effect	PVE%
Grain length (GL)						
qGL4	4	id4008430-id4009024	25.64–27.93	3.41	−0.14	3.47
qGL7	7	id7003359-id7003748	21.79–22.93	4.14	0.22	8.05
qGL8	8	id8006789-id8007301	24.21–26.84	5.37	−0.42	29.61
qGL11	11	id11007488-id11008036	21.86–23.28	4.32	−0.31	16.84
Grain width (Gw)						
qGW1a	1	id1024972-id1025983	41.12–42.57	3.18	−0.11	13.62
qGW1b	1	id1025983-id1028304	42.57–44.67	5.11	−0.10	11.58
qGW7	7	id7002051-id7002105	12.23–13.42	3.69	−0.01	0.25
qGW8	8	id8006789-id8007301	24.21–26.84	11.19	0.10	9.77
Length width ratio (LRW)						
qLWR1	1	id1000556-id1001073	6.57–11.74	4.56	0.10	2.88
qLWR8	8	id8006789-id8007301	24.21–26.84	14.55	−0.31	21.90
qLWR9	9	id9001297-id9001352	5.66–5.87	3.45	−0.16	5.91
1000 grain weight (TGW)						
qTGW1	1	id1024972-id1025983	41.12–42.57	5.49	−2.27	32.52
qTGW3	3	id3013765-id3014401	30.02–31.15	3.05	−1.21	8.83
qTGW8	8	id8006789-id8007301	24.21–26.84	5.28	0.59	2.01

A positive additive effect means Sadri alleles increasing the trait value.

Chr, chromosome; LOD, logarithm of odds

PVE%, phenotypic variation.

## Discussion

The correlation coefficient analysis among the four studied traits showed a significant correlation among each other at significance levels of 0.001, 0.01, and 0.05 (Table 1). The negative correlation of the grain length with grain width while its positive correlation with grain length–width ratio are in accordance with previous reports by Bai et al. (2010) and Wan et al. (2006). Interestingly some other studies like Liu et al. (2010) have shown the contradictory correlation between GL and GW, indicating toward a complex relationship between the two trials. Our study found that grain length showed a positive but minor correlation with grain weight (*p* < 0.05). The results showed grain width had a negative effect on the grain length–width ratio, while positive correlation with 1,000 grain weight; similar results were reported previously (Liang-Qiang et al., 2006; Wan et al., 2008). The length/width ratio showed a significant negative correlation with 1,000 grain weight, in accordance with previous observations reported by Liu et al. (2010). The result showed that grain size (length and width) is an important agronomic trait for rice breeding because it indirectly influences the grain yield by affecting the grain weight. Interestingly, based on the significance level, grain width contributed more in 1,000 grain weight in this population than the length.

In this study, an F_2:3_ population of 94 individuals derived from IR64 (indica) and Sadri (aromatic) from Iran was used for mapping with high-throughput SNP markers. The rice SNP set of 384-plex named RiceOPA (oligo pool assay) 2.1 made for indica/indica comparison and RiceOPA3.1 (indica/japonica) found 213 and 190 polymorphic SNPs, respectively. RiceOPA 2.1 was used for genotyping of F_2_ population because indica and Aus are closer than japonica and aromatic reported by Thomson et al. (2012). The genetic map covering the 1783.4-cM genomic region was constructed with an average marker interval of 8.37 cM which is below the minimum required interval (20 cM) for QTL mapping (Lander and Botstein, 1989; Bogdan and Doerge, 2005).

A genetic linkage map of the 1635.9-cM genome region was constructed using 164 SSR markers for RIL population derived from Nanyangzhan and Chuan7 parents by Bai et al. (2010). Liu et al. (2010) constructed a linkage map of 1,371.4 cM length using evenly distributed 133 SSR markers on a RIL population. The linkage map covering 2005 cM was contracted using 175 polymorphic RFLPs by Huang et al. (1997). The difference in different reported studies was found, which was due to the variation in the genetic background and recombination rate in the developed population.

Diversity analysis results confirmed the previous reports by Garris et al. (2005) that both the studied lines were genetically very distant from each other. IR64 and Sadri belong to the indica group and aromatic, respectively, as shown in Supplementary Figure S2.

Grain length and width can be simply categorized visually. The grain shape is mainly determined by the length/width ratio. Using the high-throughput BeadXpress SNP platform, 14 QTLs were detected controlling the rice grain shape, grain size, and grain weight in cross between IR64 and Sadri.

Out of four QTLs of grain length, a QTL qGL8 was not previously reported for this trait based on a comparison of genetic position and/or physical position but reported for grain breadth and grain shape by Rabiei et al. (2004) in indica/indica cross using Iran traditional varieties. The second grain length QTL qGL7 was near to qGL7-2 (Shao et al., 2010) but having a low LOD score. Two major QTLs, qGL7 (Bai et al., 2010) and qGL7-2 (Shao et al., 2010) reported on chromosome 7, were showing positive additive effects on grain length 0.30 and 0.24, respectively, in indica/japonica cross.

Four additive effect QTLs were detected for GW by using the SNP marker on chromosomes 1, 7, and 8. Two QTLs qGW1a (LOD 3.11) and qGW1b (5.11) were detected first time on chromosome 1 having a phenotypic variation of more than 11% between the interval of id1024972-id1052983 and id1052983-id1028304, respectively. These QTLs were not reported previously in this region. The QTL qGW8 was previously reported between the interval of RM256 and RM230 by using two traditional Iranian rice cultivars and also positively affect the grain breadth (Rabiei et al., 2004). Yang et al. (2021) also found a QTL of grain width at the bottom end of chromosome 8 by substitution mapping.

Three QTLs for the length/width ratio were identified on chromosomes 1, 8, and 9. The minor QTL qLWR1 explains 2.88% phenotypic variation with the LOD of 4.56 on the tip of the chromosome 1, which was previously reported by Jiang et al. (2005), using double haploid (DH) population derived from the indica (Zhenshan 97) and japonica (WYJ-2), at the same position. The QTL on chromosome 8 between marker intervals of id80006789-id8007301 had an LOD score of 14.55 with a phenotypic variation of 22.90%. A similar region was also reported by Rabiei et al. (2004) using indica/indica population. Bazrkar-Khatibani et al. (2019) also found a QTL of grain width at the bottom end of chromosome 9 by using RIL population derived from a cross of Ali-Kazemi (A) and Kadous (K) between the marker intervals of RM23904 and RM24432.

For 1000 grain weight, three QTLs were detected, out of which QTL qTWG1 on chromosome 1 explained 32.52% of phenotypic variation. The allele of IR64 at the locus qTWG1 had a major effect on 1000 grain weight. Two QTLs were detected on chromosomes 3 and 8 with 8.83% and 2.01% of phenotypic variation, respectively. Liu et al. (2010) reported two QTLs on chromosome 3, between the marker intervals RM3400–RM3646 (PVE: 24%) and RM3436–RM5995 (PVE: 19.6%) in cross between Minghui63 and Teqing (indica/indica).

Most of the QTLs found in this study were not previously reported because the previous studies for grain size and shape were performed using indica/indica or indica/japonica crossed populations. However, in this study, a population derived from long grain indica and aromatic indica varieties was used to investigate grain-related traits. The important finding of this study is a major QTL on chromosome 8 which was controlling both grain size and grain weight with a high phenotypic variation. This QTL was previously reported for grain breath and shape by Iranian scientists (Rabiei et al., 2004; Nili et al., 2017; Qiu et al., 2017), while using Iranian traditional varieties. We identified SNP alleles for grain-related traits in the genetic background of non-aromatic varieties; furthermore, these SNP markers can be used for QTL mapping studies and can be substituted by SSR or other molecular markers.

## Conclusion

QTL mapping analysis for grain size, grain shape, and grain weight by using F_2:3_ population derived from IR64 and Sadri revealed 14 QTLs for all four traits. Interestingly, one region in chromosome 8 contained QTLs for all the 4 studied traits (1 for each trait), making this a good candidate for the fine map to identify candidate genes for grain-related traits. The study generates reliable information about the SNP map and contributes for mapping QTLs in biparental population.

## Data Availability

The original contributions presented in the study are publicly available. This data can be found here: https://www.ebi.ac.uk/biostudies/studies/S-BSST885.

## Supplementary material

The Supplementary Material for this study can be found online at: https://www.frontiersin.org/articles/10.3389/fgene.2022.955347/full#supplementary-material
