# Supplementary material for: Identification of QTLs for rice grain size and weight by high-throughput SNP markers in the IR64 x Sadri population
Source: Front Genet. 2022 Aug 19;13:955347. doi: 10.3389/fgene.2022.955347 (PMC9437704; doi:10.3389/fgene.2022.955347)
Supplement: Supplementary file 2 [file DataSheet1.docx]

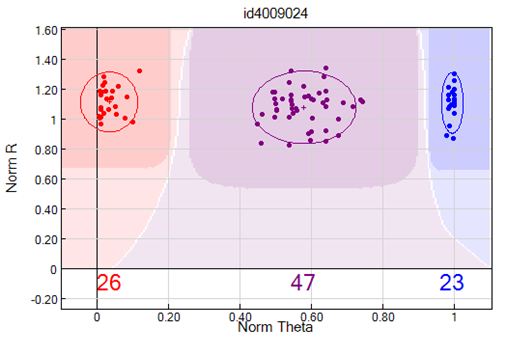


**Fig S 1.** Clusters of alleles based on the ratio of the cy3/cy5 signal (BeadXpress)


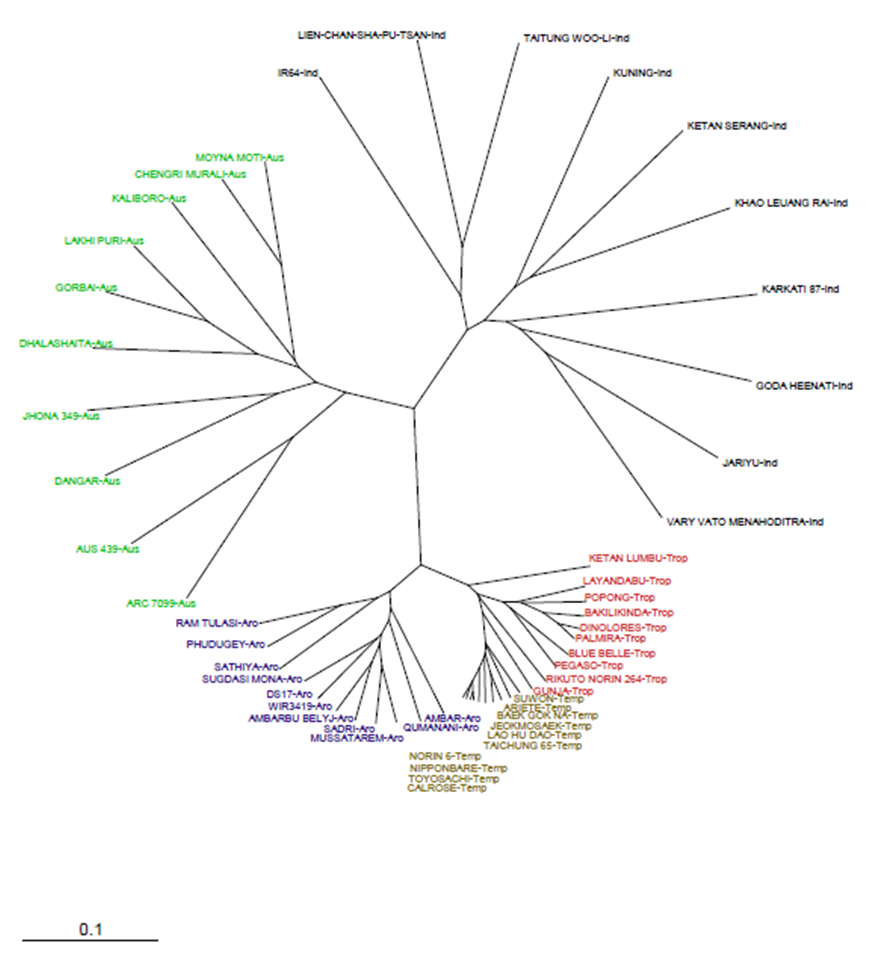


Aromatic

Indica

**Fig S 2.** Diversity analysis of 50 rice accessions (Thomson *et al.* 2012)
